# Complementary and Alternative Medicines in Northern Cyprus: Public Awareness, Patterns of Use, and Attitudes

**DOI:** 10.3390/healthcare11070977

**Published:** 2023-03-29

**Authors:** Doğa Ünlüyol, Hüseyin Gökçekuş, Youssef Kassem, Murat Tezer, Filiz Meriçli, Dudu Özkum Yavuz

**Affiliations:** 1 Department of Phytotherapy, Pharmacy Faculty, Near East University, Nicosia 99138, Cyprus; 2 Department of Civil Engineering, Civil and Environmental Engineering Faculty, Near East University, Nicosia 99138, Cyprus; 3 Energy, Environment, and Water Research Center, Near East University, Nicosia 99138, Cyprus; 4 Department of Mechanical Engineering, Engineering Faculty, Near East University, Nicosia 99138, Cyprus; 5 Arts and Sciences Faculty, Near East University, Nicosia 99138, Cyprus; 6 Department of Pharmaceutical Botany, Pharmacy Faculty, Near East University, Nicosia 99138, Cyprus

**Keywords:** Northern Cyprus, herbal medicine, herbal products, complementary and alternative medicine, common illnesses

## Abstract

**Background**: The utilization of herbal medicine (HM) as a component of complementary and alternative medicine (CAM) is increasing worldwide. Little is known about justifications for its use and the factors associated with it. This study gains insights into the use of herbal medicines in Northern Cyprus, concentrating on targets for its use, the role played by disease type, reasons for its use, and sources of information. **Methods**: A questionnaire was utilized to achieve the aim of the study. The questionnaire was distributed to a random sample comprised of people in two different regions in Northern Cyprus over a 12-week period from August to November 2020. A self-administered questionnaire was used for data collection. Moreover, qualitative research explored individuals’ decision making regarding CAM, which aimed to examine 20 patients as a context for beliefs, decision making, and dialogue about CAM. After audio-taping and verbatim transcription, the data were analyzed with qualitative content analysis. **Results**: The findings show that the majority of respondents learned about herbal products (HPs) and CAM from other patients, the Internet, friends, and family. The results indicate that almost half of the respondents were aware of how CAMs, especially herbal preparations, are used to treat common illnesses. *Fennel*, *ginger*, and *echinacea* were the most commonly used HMs, mainly for the treatment of the common cold. Furthermore, nearly 50% of the participants expressed the belief that HMs are safe, have fewer side effects than conventional medicines, and are also effective for treating minor health conditions. The prevalence was strongly associated with education level based on a Pearson Chi-square analysis. **Conclusions**: Although herbal medicines were mostly used to treat mild to moderate ailments and the participants were aware of their limitations, the combination of self-medication, inexperienced counseling, and lack of awareness of the risks of herbal medicines is potentially harmful. This is particularly important for elderly users, because although they seemed to be more aware of health-related issues, they generally used more medication than younger people. Given our finding that dissatisfaction with modern medicine is the most important reason for the preferred use of herbal medicines, government agencies, physicians, and pharmaceutical companies should be aware of this issue and should aim to create some level of awareness among users.

## 1. Introduction

Complementary and alternative medicine (CAM) refers to a variety of healthcare and medical systems, practices, and products that are not part of conventional medicine [1,2]. CAM is referred to as “traditional medicine”, according to Heinrich et al. [3]. Traditional medicine is an essential element of healthcare in many regions of the world, such as Thailand, India, Malaysia, Indonesia, China, Taiwan, Poland, Hungary, Austria, Germany, Italy, and Switzerland [3]. According to Larsen [4], several factors distinguish CAM from modern medicine, such as “modern medicine is organ-specific”, and “modern medicine prefers patients to be passive, whereas CAM requires the patient to take a highly active part”. Moreover, CAM can be categorized into five groups [5]: (a) whole medical systems, including Ayurveda, homeopathy, naturopathy, and traditional Chinese medicine; (b) mind–body techniques, including biofeedback, hypnotherapy, prayer, and relaxation techniques; (c) biologically based practices, including chelation and diet therapies; (d) manipulative and body-based therapies, including chiropractic, osteopathic manipulation, cupping, massage, moxibustion, reflexology, and gua-sha; and (e) energy therapies, including acupuncture, magnets, reiki, and therapeutic touch.

In general, the reasons for CAM’s popularity are most undoubtedly complex. They are associated with social and cultural contexts [6]. In addition, these might differ from therapy to therapy as well as from one individual to another. Several interrelated positive and negative factors interplay to determine CAM’s present level of popularity [7,8]. The positive motivating factors include perceived efficacy, perceived safety, philosophical beliefs, control over treatment, and a good patient–therapist relationship [7,8]. On the other hand, negative motivating factors are those associated with perceptions about conventional medicine, including possible serious adverse effects, poor doctor–patient relationships, insufficient time with the doctor, long waiting lists, and rejection of science and technology [7,8].

CAM is gaining popularity in many countries and among many people, with billions of dollars being spent on the practice. In the literature [9,10,11,12], around 75% of the population in France, 42% of the population in the United States, 86% of the population in Egypt, and 88% of the population in the Kingdom of Saudi Arabia were found to utilize CAM and herbal medicine (HM) for primary care. Moreover, traditional healers treat 90% of patients in Bangladesh, 85% in Myanmar, 80% in India, 75% in Nepal, 65% in Sri Lanka, and 60% in Indonesia according to a WHO survey [13].

Globally, many studies have been conducted on different populations to examine the attitudes and profiles of people who use CAM and the reason for using it [14,15,16,17,18,19,20,21,22]. Some studies have also been conducted to investigate the association between CAM use and adverse health effects [23,24,25,26]. The results from these studies demonstrated that HMs are generally utilized to treat mild to moderate diseases, and the participants were aware of their limitations. The combination of self-medication with inexperienced counseling and a lack of awareness of the hazards associated with herbal medicines can be harmful.

Moreover, according to the authors’ review, CAM is considered an integral part of the health practices in Northern Cyprus. Additionally, three studies related to this area have been conducted. For example, Dudu et al. [27] listed the plants that are utilized as traditional medicines for the treatment of diabetes mellitus in Northern Cyprus. The results showed that the use of herbal medicines was common among patients with diabetes and that some herbs may have potential drug interactions with medicines used concurrently. Christopher and Ozturk [28] determined the prevalence and determinants of CAM use among Nigerian children living in Northern Cyprus. The results demonstrated that the most widely utilized CAM products were vitamins, minerals, and herbal products. Tülek et al. [29] identified the HM products used in pregnancy in community pharmacies in Northern Cyprus. The results indicated that herbal lozenges were the most common form of herbal pharmaceuticals.

Of these studies, none have been performed in Güzelyurt (agriculture region). This study was conducted to highlight the attitudes toward and use of CAM among a random sample in two different regions, namely, Lefkoşa (urban region) and Güzelyurt (agriculture region). Therefore, the purpose of the study is to examine the use habits of HM products among the participants in Northern Cyprus, as well as their frequency and their reasons for using them. In addition, this study aims to explore the public’s views, attitudes, and experiences towards CAM, HM, and herbal products (HPs) to promote the use of safe medicines and to develop recommendations for improving public health in light of the data collected in Northern Cyprus. To the best of our knowledge, this is the first study to explore the most commonly utilized HPs during the pandemic and to determine the prevalence, reasons for utilization, and beliefs about HPs among a representative sample of the Northern Cyprus population.

## 2. Materials and Methods

### 2.1. Study Setting

To assess the public’s views, attitudes, and experiences regarding CAM, HM, and HPs, this study conducted a qualitative and quantitative analysis of data collected from an online survey and in-person interviews (face-to-face).

In the first stage, a survey was developed by a panel of experts with the participation of four community pharmacy-owning pharmacists working in NC, 5 (2 phytotherapy, 1 pharmaceutical botany, 1 public health, and 1 communication) academicians, and a nurse therapist. The authors carefully selected the expert representatives to ensure that all participants comprehensively understood CAM. The panel interview style was chosen because it allowed the interviewer to be systematic while also allowing the respondents to express their thoughts in their own words as well as to discuss common interests and experiences. It addressed the attitudes and experiences toward CAM and HM products as well as the concomitant use of HMs with modern medicines and counseling about their use.

In the second stage, participants for the face-to-face interviews were randomly selected from the public. Using the lottery method, simple random sampling was utilized to select the participants. The significance of the study topic in terms of public health was explained in detail to the interviewees to enable them to better understand the survey and increase their level of engagement. It should be noted that the interviews were held in different locations in the selected regions (Lefkoşa and Güzelyurt) to ensure diversity. Each interview lasted between approximately 30 to 45 min.

In the third stage, a cross-sectional study was carried out on local participants aged 17 years and older in two regions (Lefkoşa and Güzelyurt) of Northern Cyprus. Participants were registered by posting advertisements in public Facebook groups. The post included an introduction to CAM and HP products and their use and a survey link to a self-administered questionnaire generated using Google Forms. Furthermore, the survey link was distributed to participants who had been previously contacted during personal interviews (face-to-face). Additionally, participants were asked to share the link with their contacts on Facebook because it is the most widely used platform in Northern Cyprus.

The survey was available for 12 weeks from August to November 2020. Due to requests for anonymity, the names of the interviewees were not disclosed. Moreover, the data obtained from the participants were analyzed, which were examined separately by two qualitative research experts. The thematization and classification process was performed by taking co-coding into account. Content analysis was applied to the qualitative data.

### 2.2. Questionnaire Design

The questionnaire consisted of a total of 28 questions, including 17 questions created to measure HP use habits, 8 questions about CAM approaches, and 3 questions about seminars. Three field experts were consulted for the content and face validity of the prepared questionnaire. After the necessary corrections were made to the questionnaire, the final form was established. The self-administered questionnaire was organized as follows:

- Section 1 assessed the participant’s sociodemographic background, including gender, age, marital status, region, education, occupation, and monthly income.
- Section 2 investigated the participants’ use of HPs, their thoughts on the safety of HPs, in which situations they prefer to use HPs, in which forms they prefer HPs, the factors affecting their use of HPs, where they obtained HPs, and where they obtained information about the HPs they would use.
- Section 3 contained questions about whether the participants had tried CAM treatment methods, including aromatherapy (massage, inhalation, compress, bath), phytotherapy (treatment with HPs), apitherapy (honey, propolis, royal jelly, pollen), Ayurveda, homeopathy, ozone therapy, acupuncture, yoga, meditation, religious and spiritual healing (praying), chiropractic treatment, osteopathy, hypnotherapy (hypnosis), music therapy, hydrotherapy, cupping therapy, and leech therapy, which methods they had tried, where they learned about such treatments, for what purposes they used them, and whether they recommended the methods they used to others.
- Section 4 asked participants about their attitudes towards HMs and HPs and whether they would find public seminars useful regarding HMs and HPs to increase their knowledge about them.
- Section 5 included five open-ended semi-structured questions to obtain qualitative data. The open-ended questions were:
  ⚬ Beyond your prescribed treatment, have you tried other practices like herbs, vitamins, plant oil massage CAM, or anything else for your health problems? If yes, which kind of such practice? What is your opinion about CAM?
  ⚬ What is your attitude toward the concept of CAM? Is it beneficial? From where did you get information about CAM?
  ⚬ Do you think CAM is effective for your health problems?
  ⚬ Have you informed your doctor, pharmacist, or any other healthcare professional about the use of CAM? If not, why?
  ⚬ Are there any issues related to CAM that you would like to add?

Twenty patients who voluntarily participated in the interview were asked qualitative questions, which were prepared with input from field experts, and the interviews were recorded with the participants’ permission. The content validity of the interview forms was ensured in line with input from four field experts. At the end of the semi-structured interviews, the reliability of the qualitative data obtained from the research was ensured through coding categorization and thematization processes separately by two expert analysts.

### 2.3. Data Analysis

Data were exported in a CSV file from Google Forms and then processed so it could be utilized by IBM SPSS STATISTICS V21 for data analysis. A Pearson Chi-square analysis *p*-value < 0.05 was used to reveal the indicated statistical difference among categorical parameters.

### 2.4. Ethical Consideration

To collect participant data, ethical approval was obtained from the Near East University Ethics Committee (NEU/2020/83-1151). In addition, all interviews were conducted after the verbal and written consent of the participants had been obtained.

## 3. Results

### 3.1. Participants’ Data

The participants were selected from different regions to understand the impact of the region on the results. Lefkoşa was selected due to the high density of population and workplaces, while the Güzelyurt region was chosen to provide better population diversity in terms of its more rural location and intensive engagement in agriculture. The distribution of participants was as follows: 246 (68.5%) in Lefkoşa and 113 (31.5%) in Güzelyurt, as shown in Table 1. Moreover, the participants were chosen from different genders and levels of education to understand how these variables impacted the results. As shown in Table 1, 225 (63.4%) of the participants were females and 288 (81.4%) had graduated from a university or higher education program. Additionally, with regard to age, 44.4% were in the 17–35 age group, 24.9% were in the 36–45 age group, and 30.7% were in the 46–84 age group. Most of the participants had a moderate income, as indicated in Table 1.

### 3.2. Participants’ Awareness of CAMs

A total of 63.0% (*n* = 226) of participants had tried CAM approaches, and the most preferred method was phytotherapy with 50% (*n* = 113), followed by aromatherapy with 44.2% (*n* = 100), and apitherapy with 37.6% (*n* = 85). While 38.7% (*n* = 139) of the participants thought that CAM is helpful, 4.5% (16) believed that it is not at all useful. When the respondents were asked where they had learned and tried CAM methods, 27.9% said they were advised by a doctor; 22.8% said they received advice from family, friends, or neighbors; 22.6% were advised by a pharmacist; 21.2% had seen information on the Internet or television and tried it; and 4.7% said they tried it because they had seen information in a newspaper or magazine. When respondents were asked about the number of places providing complementary and alternative treatment services and the adequacy of the educational status of the practitioners providing CAM treatment services, 53.8% (*n* = 193) stated that the number of places was insufficient. In contrast, 30.4% of the same participants thought that the knowledge of CAM providers is insufficient. When the respondents were asked why they used CAM, 40.7% (*n* = 146) used it as an immune booster, followed by 22% (*n* = 79) for sleeping disorders stress, 20.9% (*n* = 79) for burnout syndrome, and 20.6% (*n* = 74) for lumbar hernia, rheumatism, musculoskeletal disorders, etc., as listed in Table 2.

### 3.3. Use of and Public Attitudes towards HPs

**Figure 1:**
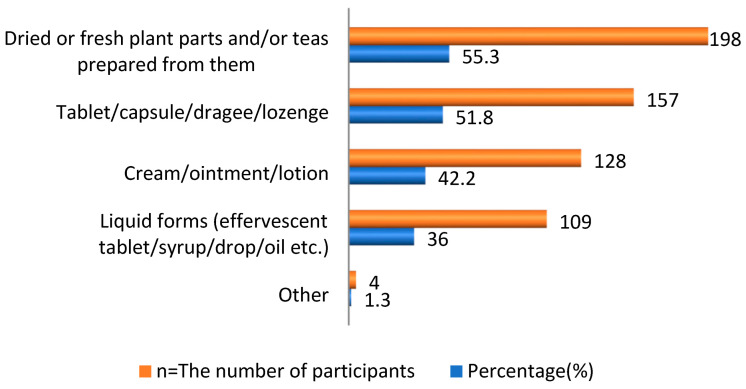
The forms preferred by users of HPs.

Considering the use of HPs, 303 participants (84.40%) had used at least one herbal product. As for the preferred format of the HPs, 157 (51.8%) participants preferred solid forms, such as tablets, capsules, dragees, or lozenges, and 198 (55.3%) participants preferred dried or fresh plant parts and/or tea prepared from them as shown in Figure 1. The most preferred HPs were fennel (*n* = 208, 57.9%), ginger (*n* = 181, 50.4%), and echinacea (*n* = 146, 40.7%), as indicated in Table 3.

It was observed that the participants’ main purpose when using HPs was to prevent sickness and protect health (*n* = 251, 69.9%). While the number of participants who used HPs to cure an existing disease was 125 (34.8%), the number of those who used HPs to prevent the symptoms of their disease was 132 (36.8%). The majority of the respondents thought that HPs are natural, harmless, and chemical-free (*n* = 187, 52.1%) or that the risks of side effects, drug interactions, and allergies are very low (*n* = 95, 26.5%), as shown in Table 4.

**Figure 2:**
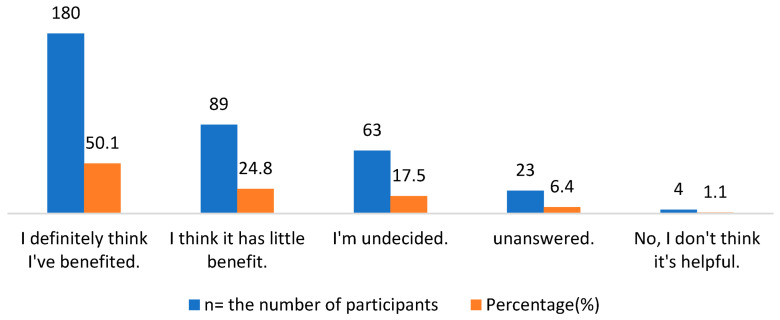
The extent to which the respondents benefited from HPs according to their intended use.

**Figure 3:**
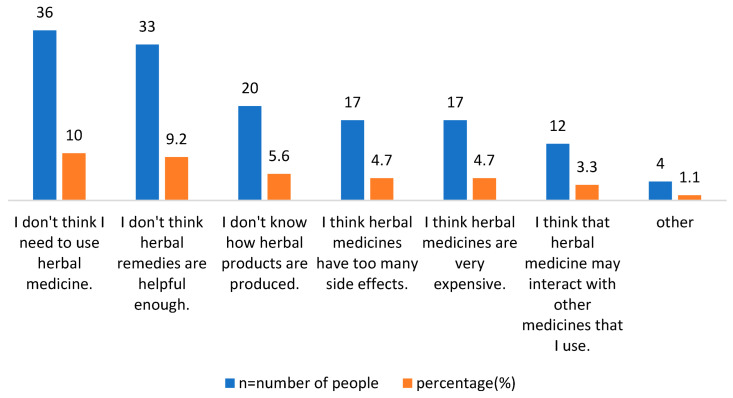
The reasons for the respondents not using herbal products.

Among the respondents, 180 participants (50.1%) thought that the HPs they use are beneficial according to their intended use, while 89 (24.8%) thought they provide minimal benefit, as illustrated in Figure 2. Moreover, the authors asked the respondents about their reasons for not using herbal products. As shown in Figure 3, 10% of the participants thought that they do not need to use herbal products, 9.2% thought that herbal products are not useful enough, and 5.6% expressed reluctance to take such supplements because they do not know the production conditions of herbal products. Some 4.7% of respondents thought that products are very expensive, and 4.7% of them said they do not use such products because of the possible side effects of herbal products. Finally, it was observed that 3.3% of the participants did not use such products because they thought that the herbal products might interact with the conventional drugs they were currently using.

**Figure 4:**
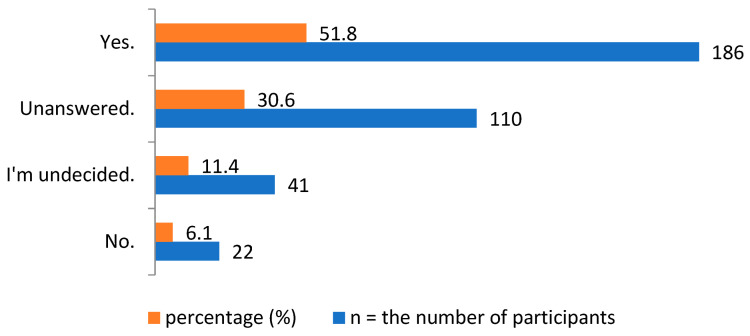
Respondent’s opinions on whether they would take HPs when a doctor or pharmacist recommends those that are beneficial for the patient’s disease in addition to conventional medicine.

The number of participants with chronic diseases among the survey participants was 107 (29.8%). When asked if they would inform their doctor/pharmacist about the HPs they use, of the 67 participants who used conventional medicine as well as HPs for their chronic disease, the majority (*n* = 43, 64.2%) said they would do so (Table 5). The respondents were asked if they would use HPs in addition to conventional medicine if recommended by their doctor or pharmacist to. While 186 participants (51.8%) said yes, 22 participants (6.1%) stated that they would not use them, as shown in Figure 4.

Furthermore, when the respondents were asked whether herbal medicines/products are as effective as the conventional medicines currently on the market in the treatment of certain diseases, 55.2% (*n* = 198) of the respondents said they were partially effective, while 25.1% (*n* = 90) said they were effective, and 1.9% (*n* = 7) stated that they are not effective at all. It was observed that the respondents mostly preferred to use HPs in cases, such as cold, flu, cough, etc. (*n* = 278, 77.4%), and to strengthen the immune system (*n* = 234, 65.2%), as indicated in Table 6.

When asked about the most effective factors in the use of HPs, 61.6% of the participants said that a doctor’s advice (*n* = 221) and 56.5% cited a pharmacist’s advice (*n* = 203) as the most important factors, while the Internet (*n* = 139, 38.7%) and neighbor/friend/family recommendations (*n* = 113, 31.5%) were also found to be other effective factors.

After deciding to use the product, 71% (*n* = 255) of the participants said that they would consult their pharmacist about the product when asked about the methods they would use to collect information, while 53.8% (*n* = 193) said they would consult their doctor, and 52.9% (*n* =190) stated they would gather information from the Internet. The proportion of those who said they would ask their neighbors and/or friends who had used the product about their experience was 22.3% (*n* = 80), while the rate of those who said they would use it without conducting any research was 1.9% (*n* = 7).

When asked which factors were important when buying HPs, 71% (*n* = 255) cited their pharmacist’s suggestions, 65.5% (*n* = 235) said their doctor’s suggestions, 47.6% (*n* = 171) looked at the manufacturer brand HPs, 20.9% (*n* = 75) said the price, and 8.1% (*n* = 29) said advertisements on the Internet and television. Considering the distribution of HP use according to age, gender, education level, and income level, people with a master’s degree or higher education level preferred fewer HPs than those whose with less education, 78.5% to 88.16%, respectively. Individuals between the ages of 36and 45 were the most likely to use herbal products (88.8%). However, when the effect of age, education level, gender, and income status on the use of HPs was examined, no statistically significant difference was found (Table 7).

In addition, Chi-square tests were performed for the comparisons made in Table 7. Significant associations (*p*-value < 0.05) were found between education level and HP use status. Additionally, there was no significant difference between age and HP use (χ^2^ = 2.665, *p* = 0.264 > 0.05) as patients used HPs regardless of age. As a result of the Chi-square test, no significant difference was found between the gender variable and HP use status (χ^2^ = 0.272, *p* = 0.602 > 0.05) as patients used HPs regardless of gender. There was no significant difference between the income variable and HP use status (χ^2^ = 5.515, *p* = 0.238 > 0.05). In other words, patients used HP regardless of their income level.

Moreover, Table 8 presents responses regarding the average monthly budget for the herbal products used. It was found that women spent more money than men, and the difference was statistically significant (χ^2^ = 6.575 a, *p* = 0.037 > 0.05). As for the effect of education, 44.7% of individuals with graduate and higher education levels spent TL 150 or more on herbal products, while this rate was 30.2% for individuals with university degrees, and only 23.7% for those with a high school education. The difference was found to be statistically significant (χ^2^ = 14.125 a, *p* = 0.007 > 0.05).

### 3.4. Public Attitude towards Possible Future Seminars

Finally, the participants were asked whether holding a seminar on HPs and CAM treatments would be useful and whether they would participate if such a seminar was held. While 80.8% (*n* = 290) of the participants stated that they would find it useful, 56.8% (*n* = 204) of the same participants stated that they would attend a seminar on this subject.

### 3.5. Qualitative Findings of the Study-Interview Analysis

In this section, the findings were interpreted by obtaining study participants’ on herbal products and complementary and alternative medicines from a qualitative point of view.

The interview content analysis identified three main themes: (a) familiarity and understanding of CAM; (b) attitudes and perceived benefits; and (c) disclosure to the physician. Each topic with illustrative excerpts from patients’ texts is described below.

Theme 1: Familiarity and understanding of complementary and alternative medicine.

Patients were asked about their use of any alternative therapies to the approved standard of care.

“I took my treatment as my doctor said, but I prefer to use some herbal and vitamins with it because they make me feel better and they are good… I also tried some dietary supplements to improve my health in general”.

“Yes, sometimes I drink herbs and lemon juice when I feel that my pressure is high. Also, I do use CAM from time to time and I feel better after doing it”.

Only a small number of patients claimed that they adhere to the therapy advised by the doctor and have never attempted any alternative method; nevertheless, they said they follow a diet restriction program.

“No, I’ve never attempted anything other than the doctor-prescribed therapy... Because they have not been evaluated and I am unsure of the potential consequences of using them, I do not trust products like herbs or CAM therapies. I altered my diet and took the required medications.”

Some patients explained why they were seeking such therapies. The majority cited affordability, safety, religious difficulties, and advice from friends.

“My friend advised me to use one of the CAMs to treat hypertension, and when I tried it, I felt good without the need to use medicines”.

“Natural products are not manufactured by a human; they have been found in nature for our benefit since the beginning of life. They are like our food, they are also cheap and not like manufactured drugs which are very expensive and may result in harm to the patients”.

The interviews revealed that respondents in this study were unfamiliar with the term CAMs; instead, they knew this sort of therapy as herbal medicine.

“No, I’ve never heard of this name before, but I know this therapy is known as herbal medicine. No, I was unaware of this before... But, I have heard about alternative medications on television and believe they are the same as herbal remedies”.

Theme 2: Attitudes and perceived benefits of complementary and alternative medicine.

The majority of individuals utilized a range of CAM products for hypertension. Among the most frequently suggested items were garlic, lemon, vitamins, and nutritional supplements. However, the data do not indicate the initial product used by each participant, as the majority of them had utilized many types of CAM at the same time and since their diagnosis.

“I have used different things since I found out I have hypertension… I drink lemon; also, I take some food supplements with garlic, ginger, and vitamins…”.

Pharmacists and friends were the main sources of information regarding CAM. Furthermore, the respondents said that they could easily obtain such products from the markets, herbalists, and community pharmacies at reasonable prices.

“I got them from the pharmacy… I prefer to ask the pharmacist about them because he knows more about the medicines and herbs”.

“Herbal medicine has been known since ancient times and when I need to use it or need any advice, I ask older people or the pharmacist sometimes”.

Patients perceived that CAM therapy is effective in lowering high blood pressure based on regular use. Another noted perception was that CAM is a safe practice and free from any side effects.

“Yes, it is an effective treatment and gives good results, but you have to take it continuously… If you leave it for a long time, maybe you will not get any benefit from it. It needs time to lower the blood pressure”.

“I think they are safe, without any harmful effects… Our religion encourages us to do it. Reflexology is also good and safe as I have tried it before without any bad results”.

Theme 3: Communication with the physicians and other medical staff.

The last theme suggested that respondents did not disclose CAM practices to their doctors. In most cases, the respondents said they tend to use CAM despite their standard prescribed treatment. Reasons for nondisclosure were short counseling time, fear of the doctors’ anger, and lack of CAM recognition.

In this study, CAM users said they preferred to consult their pharmacist about CAM-related information. “I buy the herbs from the pharmacy and ask the pharmacist about the preparation and use. I think the pharmacist is the best person to obtain information about the treatment and herbal medicines because drugs were originally made from herbs”.

“Concerning CAM, I learned about it from TV shows and from reading about it on the Internet. I sometimes contact the elderly since they have experience dealing with it”.

## 4. Discussion

Based on a self-administered questionnaire and qualitative interviews, this study investigated the factors and reasons for consumers’ use of HM. The first important finding is that most users of HMs are female. This result is supported by previous studies [18,30,31,32,33,34]. Furthermore, based on the results and compared with similar studies, HP use was found to be vary from country to country [35]. For example, 66.8% of the population in Nigeria [36], 63.5% of the population in Kuwait [37], 39.2% of the population in Turkey [31], and 33.9% of the population in Malaysia [33] have used HPs. These findings may be due to differences in cultural and health systems in countries or the perception of HPs in other studies, but more studies on this topic are needed in the future to clarify this issue.

Furthermore, the participants revealed that the primary purpose of utilizing HM was to treat an illness, whereas promoting health and preventing an illness were less important. Likely, the latter two features are better addressed by dietary supplements or other kinds of complementary and alternative medicine (CAM) rather than by HM [37,38]. It may also indicate that people only engage in health-oriented behavior (such as using drugs to avoid illness or promote health) when they are seriously threatened by a health condition, not when they are healthy.

Another important finding is that 71% of the population consulted their pharmacist to gather information after deciding to use HPs, whereas 53.8% of them would consult their doctor, and 52.9% of them gather information over the Internet. While the rate of those who said they ask their neighbors and/or friends was 22.3%, the rate of those who said they would use it without doing research was only 1.9%. Compared to similar studies in Germany and the United Kingdom, it was found that Germans get information about HPs from the Internet (68.2%), pharmacists (54.2%), and doctors (37.7%), whereas people in the UK obtain information from books (57%); the Web (53%); friends, colleagues, and neighbors (51%); health practitioners (42%); and family (33%), with less relying on workshops and courses (22%) and TV/radio (17%) [35,39]. These findings are important in that they show that the people in NC trust pharmacists and doctors more than those in Germany and the UK do.

Additionally, the results indicate that the participants give more importance to the advice of their pharmacist (71%) than the price (20.9%), advertisements on the Internet (8.1%), or the company/brand (47.6%) when purchasing a product. The high rate of consultation with a pharmacist to obtain information and the high level of trust in the pharmacist when purchasing products may be due to the public’s trust in the pharmacist’s knowledge and experience about HPs and the ease of access to the pharmacist. It was found that 73.3% of pharmacists were readily available in their pharmacy for advice at any time of the day [40]. Those who participated in the survey mostly preferred to use HPs in cold, flu, cough, and similar situations (77.4%) and to strengthen their immune system (65.2%) In addition, among those who had tried CAM methods, the most frequently cited reason for use was as an immune booster (40.7%), and the most preferred HPs were fennel (57.9%) and ginger (50.4%). In addition, a study was conducted with 508 participants in Vietnam at the same time as this study that examined the use of HPs during the COVID-19 pandemic, and the findings indicated that 49% of respondents used HPs to treat symptoms of common diseases [41]. The most commonly used herbal product was ginger (*Zingiber officinale Rosc*.) with 79.1%, and HPs were mostly used in the treatment of sore throat (62.2%), cough (60.6%), nasal congestion (41.4%), and fever (35.7%) [41]. The most probable reason for these findings is that the survey was carried out during the COVID-19 pandemic when the disease was active, no vaccine had been approved, and no conventional drugs were available to provide treatment for COVID-19. The abovementioned factors could explain why people try to use alternative treatments instead of conventional treatments as immune boosters and for cold symptoms. These findings also align with a study that investigated the use of phytotherapy and diet therapy in the Balkan Peninsula during COVID-19 [42]. Another important finding is that the majority of study participants (52.1%) thought that HPs were harmless or had very few side effects, while 26.5% thought that risks of side effects, drug interactions, and allergies were very low. While 13.1% of study participants thought the risks of side effects, drug interactions, and allergies are similar to the drugs in modern medicine, only 1.9% thought that the risks are too high. This finding is similar to similar studies in Serbia, where 73.3% of the participants thought that HPs are harmless [43], the UK, where 71% thought that HPs were safe [42], and Vietnam, where 70% of respondents believed that HPs were safe, had fewer side effects than conventional drugs, and were effective in treating minor health conditions [44]. This is worrisome, considering that the risks of HPs interacting with conventional medicines and side effects need to be considered and therapeutic indices of some HPs, are very narrow. According to the results, patients use HP regardless of their gender, age, and income level, findings that agree with previous studies [30,45].

Moreover, the study showed that 84% of participants used HPs during the pandemic because they believed that HPs would significantly boost their immunity. These results are supported by previous scientific studies. For instance, Nugraha et al. [23] found that people believed that using HPs can prevent or even cure COVID-19. Nilashi et al. [46] found that using CAMs would be an effective way of boosting the immune response against infections and preventing and/or treating COVID-19. Panyod et al. [47] concluded that dietary therapy and herbal medicine can be considered potentially effective ways of preventing and/or treating SARS-CoV-2 and COVID-19. Liu et al. [48] concluded that HP (*Lianhuaqingwen*) combined with traditional therapy appeared to be more effective for patients with mild or normal COVID-19. In contrast, prepandemic studies showed much lower rates of HP use in Northern Cyprus [27,28,29]. 

## 5. Limitations

Although the results of the current study were obtained using a simple but effective online survey and in-person interviews during the COVID-19 pandemic, this study has some limitations that may be addressed in future studies.

First, an online survey platform was utilized to reach as many participants as possible in a short time. However, this may bias recruitment to younger individuals

Second, compared to data obtained in an interview-based setting, a self-administered questionnaire may have led to bias in some of the data, as the respondents may have increased or decreased their use of HPs and CAM. However, in the context of the COVID-19 pandemic, online-based and self-administered platforms were essential because they facilitated data collection that would otherwise not have been possible due to social distancing and mobility restrictions.

Third, the use of electronic questionnaires may not have sampled the population evenly. Individuals who were illiterate or did not have access to the Internet and social networks were underrepresented in these methods, making it difficult to generalize the results. Therefore, the study may need to be repeated to include communities with different educational levels and access to the media.

Finally, this study did not explore the efficacy and safety of HMs for treating COVID-19.

## 6. Conclusions

This study is important in that it is the first study to measure HP and CAM use in people in Güzelyurt and Lefkoşa. It was found that patients used HP regardless of gender, age, and income level. Although HP use in Northern Cyprus is high, people do not have sufficient information about the harms of HPs. To overcome this potential concern and prevent it from becoming a general healthcare issue, it is crucial to raise public awareness. This can be achieved by organizing public seminars, launching public awareness-raising advertisements, and distributing brochures so that the Northern Cyprus population can increase their understanding of the side effects, drug interactions, and allergy risks of HPs. In addition, considering the trust that the Northern Cyprus people have in their pharmacists and the ease of access to such pharmacists, it is very important that pharmacists regularly attend training and actively participate in public awareness activities to keep their knowledge up-to-date. In addition, healthcare professionals need to consider patients’ perceptions and thought processes surrounding CAM to deliver effective patient-centered healthcare. These actions will be even more important in the future.

This study is the first known qualitative study developed in Northern Cyprus to quantify perceptions of CAM and HPs among the general population. Previous studies on CAM were quantitative and provided data on the types and frequency of CAM use. This study revealed the perceptions of CAM in the public/patients. The most common perception was related to CAM’s effectiveness and safety. Overall, participants perceived a lack of side effects, effectiveness, and naturalistic characteristics. This study reveals the significance of promulgating CAM products and herbal products. Moreover, it indicates that natural products constitute the most popular type of CAM.

## 7. Recommendations

In the current study, the respondents stated that they were utilizing HMs for a variety of conditions without any professional supervision, which could expose them to harmful side effects and drug interactions if used with modern medicines. Therefore, doctors and pharmacists should inform patients about HMs’ efficacy and negative effects using evidence-based information, particularly when writing prescriptions and delivering medications. Furthermore, patient counseling and education are necessary to augment awareness about HMs use. Accordingly, this study should be replicated in other regions in Cyprus with a large number of respondents in the future. Finally, governmental regulation is also essential to ensure the safe use and distribution of CAMs and HPs.

## Figures and Tables

**Table 1:** The respondent’s distribution and their demographic data.

Survey Questions	Response Options	*n* (%)
Region	Lefkoşa	68.5
	Güzelyurt	31.5
Gender	Female	63.4
	Male	36.6
Age	17–35 years	44.4
	36–45 years	24.9
	46–84 years	30.7
Education	Primary school	1.4
	Middle school	1.4
	High school	15.8
	Bachelor’s degree	50.8
	Master’s degree	18.6
	Doctorate	11.9
Income	<3500 TL	12.7
	3500–5000 TL	14.4
	5000–7500 TL	29.4
	7500–10,000 TL	22.8
	>10,000 TL	20.7

**Table 2:** The participants’ purpose for using CAM techniques.

Variables	Response, *n* (%)
Immune booster	146 (40.7)
Sleeping disorders	79 (22)
Stress, burnout syndrome	75 (20.9)
Lumbar hernia, rheumatism, musculoskeletal disorders, etc.	74 (20.6)
Acne and skin blemishes	59 (16.4)
Migraine	47 (13.1)
For weight control/slimming	45 (12.5)
Depression	36 (10)
Blood pressure	25 (7)
To quit smoking	24 (6.7)
Cardiac circulation, varicose veins, etc.	17 (4.7)
Cancer protective	16 (4.5)
Other	15 (4.2)
Healing diabetic wounds	10 (2.8)
Among the chemotherapy treatments for cancer	9 (2.5)

**Table 3:** Usage rates among herbal product/drug users.

Herbal Medicines	Response, *n* (%)
Fennel *(Foeniculum vulgare)*	208 (57.9)
Ginger *(Zingiber officinale)*	181 (50.4)
Purple coneflower *(Echinacea purpurea)*	146 (40.7)
Aloe vera *(Aloe barbadensis)*	136 (37.9)
Curcumin *(terra merita)*	110 (30.6)
Garlic *(Allium sativum)*	107 (29.8)
Rosehip *(Rosa canina* L.*)*	71 (19.8)
Artichoke *(Cynara scolymus)*	60 (17.0)
Passionflower *(Passiflora incarnate)*	47 (13.1)
Senna *(Cassia angustifolia)*	46 (12.8)
Hawthorn *(Crataegus monogyna)*	46 (12.8)
Black elderberry *(Sambucus nigra)*	46 (12.8)
Grape seed *(Vitis vinifera)*	46 (12.8)
Maidenhair tree *(Ginkgo biloba)*	45 (12.5)
Cranberry *(Vaccinium Oxycoccus)*	44 (12.3)
Horse chestnut *(Aesculus hippocastanum)*	35 (9.7)
Slimming tea	31 (8.6)
Korean ginseng *(Panax ginseng)*	30 (8.4)
Weight control herbal product (capsule, tablet)	22 (6.1)
John’s wort *(Hyperıcum perforatum)*	20 (5.6)
Other	12 (3.3)
Milk thistle *(Silybum marianum)*	10 (2.8)
Evening primrose *(Oenothera biennis)*	9 (2.5)
Valerian *(Valeriana officinalis)*	6 (1.7)
Red clover *(Trifolium pratense)*	4 (1.1)

**Table 4:** The attitude of participants toward the safety of the use of HMs.

Variable	Response, *n* (%)
The risks of side effects, drug interactions, and allergies are very low.	95 (26.5)
They are natural, harmless, and chemical-free.	187 (52.1)
The risks of side effects, drug interactions, and allergies are similar to drugs in modern medicine.	47 (13.1)
The risks of side effects, drug interactions, and allergies are very high.	7 (1.9)
Undecided about the safety of herbal remedies.	27 (7.5)
Do not know.	26 (7.2)

**Table 5:** Respondents opinions on whether those who are currently using chemical drugs for their chronic disease would inform their doctor/pharmacist about their concurrent HP use.

Variable	Response, *n* (%)
I wouldn’t say for sure as it is a herbal supplement	3 (4.5)
I don’t think I would say.	8 (11.9)
Sometimes I inform them.	13 (19.4)
I will inform them.	43 (64.2)

**Table 6:** The situations in which the respondents preferred to use HPs.

Herbal Medicines	Response, *n* (%)
Cold, flu, cough, etc.	278 (77.4)
In strengthening the immune system	234 (65.2)
Skin health	140 (39.0)
Sleep problems	132 (36.8)
For low energy	116 (32.3)
Urinary tract disorders	78 (21.7)
As an antidepressant	72 (20.1)
In joint ailments	65 (18.1)
For slimming/weight control	61 (17.0)
Liver support	55 (15.3)
In pre-menstrual disorders	47 (13.1)
In cardiovascular health	46 (12.8)
In menopausal complaints	29 (8.1)
As an aphrodisiac/stimulant	22 (6.1)
In prostate disorders	15 (4.2)
Other	3 (0.8)

**Table 7:** Distribution of HP use by age, gender, education level, and income level (Question: Have you ever used HPs before?).

Variable	Response, *n* (%)		𝜒^2^ (df)	*p*-Value
	Yes	No		
Age Range [Year]			2.665	0.264
17–35	136 (85.5)	23 (14.5)		
36–45	79 (88.8)	10 (11.2)		
46–84	87 (80.6)	21 (19.4)		
Gender			0.272	0.602
Female	193 (85.8)	32 (14.2)		
Male	108 (83.7)	21 (16.1)		
Education level			6.485	0.039 *
High school and below	56 (84.8)	10 (15.2)		
University	160 (89.4)	19 (10.6)		
Master’s degree and above	84 (78.5)	23 (21.5)		
Income rate			5.515	0.238
<3500 TL	38 (86.4)	6 (13.6)		
3500–5000 TL	46 (92.0)	4 (8.0)		
5000–7500 TL	80 (79.2)	21 (20.8)		
7500–10,000 TL	70 (86.6)	9 (11.4)		
>10,000 TL	61 (84.7)	11 (15.3)		

* *p*-value < 0.05.

**Table 8:** Distribution of HP use by age, gender, education level, and income level (Question: What is the average monthly budget for the herbal products you use?).

Variable	Response, *n* (%)			𝜒^2^ (df)	*p*-Value
	0–50 TL	50–150 TL	over 150 TL		
Age Range [Year]				2.999	0.558
17–35	53 (35.8)	48 (32.4)	47 (31.8)		
36–45	22 (27.8)	25 (31.6)	32 (40.5)		
46–84	35 (35.7)	34 (34.7)	29 (29.6)		
Gender				6.575	0.037 *
Female	64 (31.2)	62 (30.2)	79 (38.5)		
Male	46 (39.0)	43 (36.4)	29 (24.6)		
Education level				14.125	0.007 **
High school and below	17 (28.8)	28 (47.5)	14 (23.7)		
University	60 (35.5)	58 (34.3)	51 (30.2)		
Master’s degree and above	32 (34.0)	20 (21.3)	42 (44.7)		
Income rate				14.729	0.065
<3500 TL	15 (36.6)	15 (36.6)	16 (39.0)		
3500–5000 TL	14 (29.2)	22 (45.8)	12 (25.0)		
5000–7500 TL	37 (39.4)	26 (27.7)	31 (33.0)		
7500–10,000 TL	19 (26.8)	18 (25.4)	34 (47.9)		
>10,000 TL	23 (37.1)	22 (35.5)	17 (27.4)		

* *p*-value < 0.05; ** *p*-value < 0.01.

## Data Availability

Data are available from the authors upon reasonable request.
